# Supplementary material for: Fibroadipogenic progenitors are responsible for muscle loss in limb girdle muscular dystrophy 2B
Source: Nat Commun. 2019 Jun 3;10:2430. doi: 10.1038/s41467-019-10438-z (PMC6547715; doi:10.1038/s41467-019-10438-z)
Supplement: Supplementary file 1 — Supplementary Information [file 41467_2019_10438_MOESM1_ESM.docx]

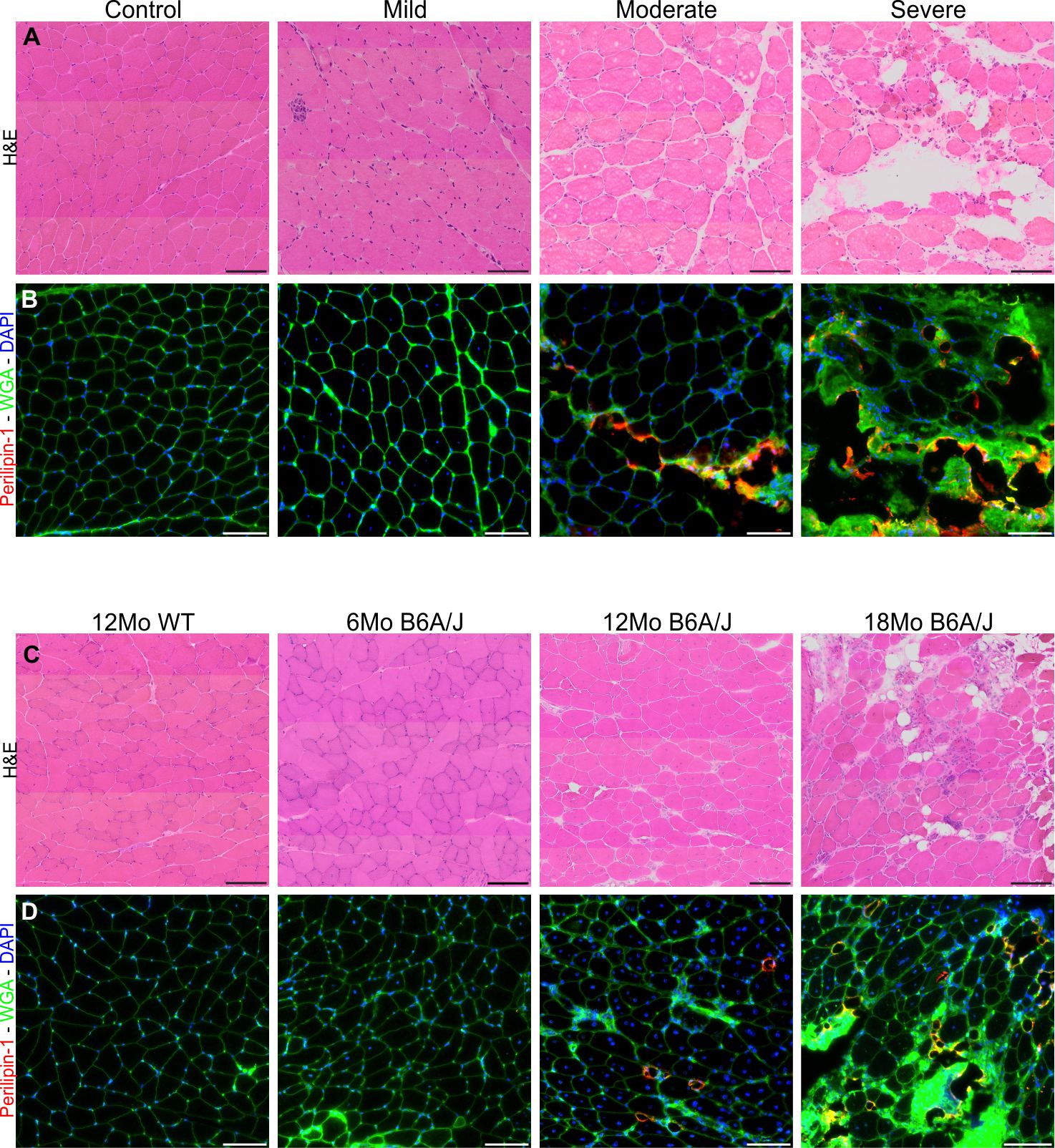


**Supplementary Figure 1: Muscle histopathology and extracellular lipid deposition increase with clinical severity.** (**A**) Hematoxylin and eosin staining of LGMD2B biopsies demonstrating increased muscle pathology with increasing clinical severity. (**B**) Perilipin-1 labelling from **Fig. 1B**, co-stained with wheat germ agglutinin to delineate myofiber membranes and demonstrate the extracellular lipid formation in LGMD2B patient muscle. (**C**) Hematoxylin and eosin staining of B6A/J gastrocnemius demonstrating increased muscle pathology with increasing age. (**D**) Perilipin-1 labelling from **Fig. 1J**, co-stained with wheat germ agglutinin to delineate myofiber membranes and demonstrate the extracellular lipid formation in B6A/J gastrocnemius with advancing age. Scales = 100µm.


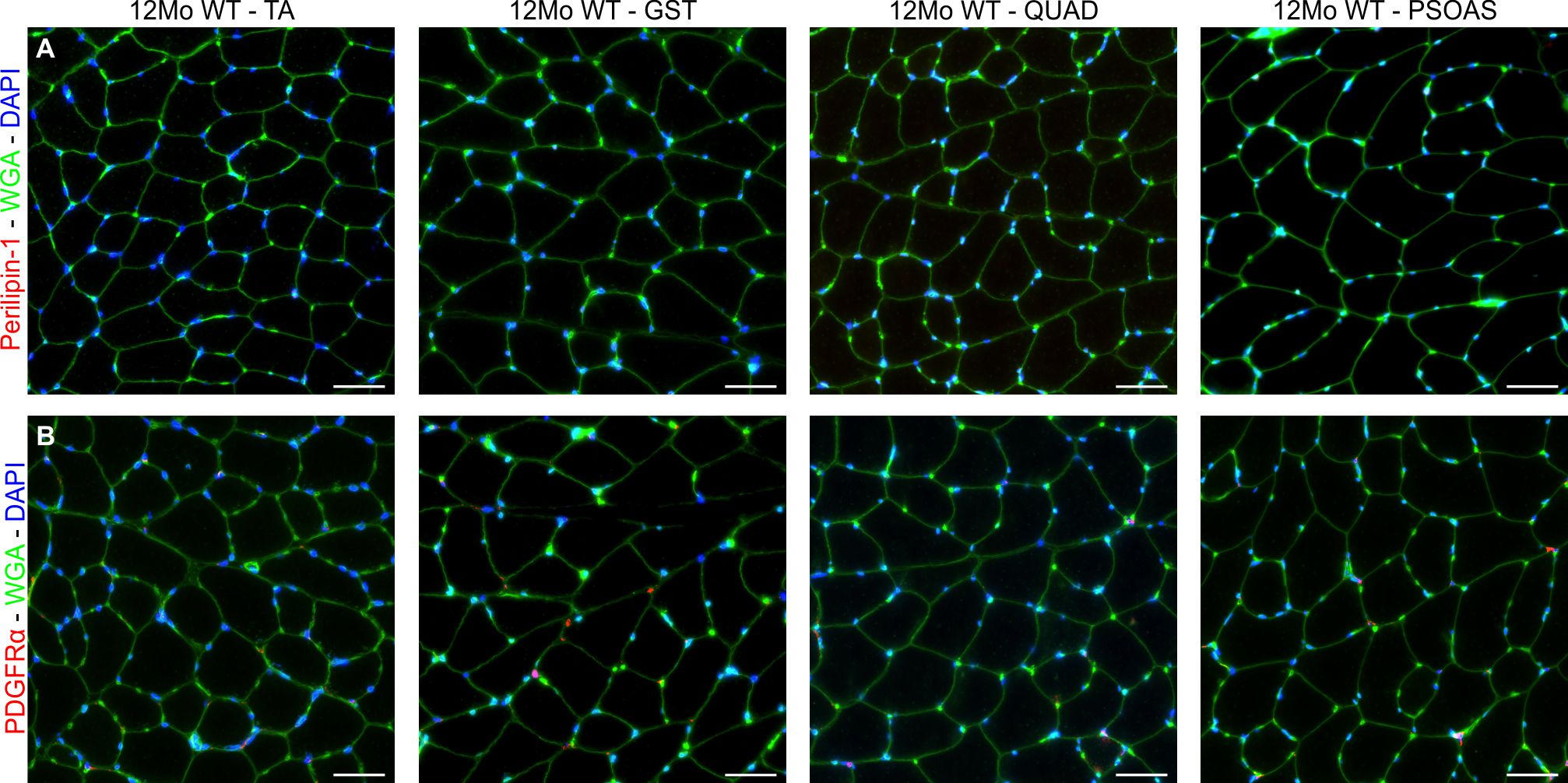
 **Supplementary Figure 2: Extracellular lipid formation and FAP accumulation is absent in WT muscle.** (**A**) Perilipin-1 staining of tibialis anterior, gastrocnemius, quadriceps and psoas of 12Mo WT mice shows the absence of extracellular lipid across all muscles. (**B**) PDGFRα staining from 12Mo WT muscles shows only the presence of muscle resident FAPs (individual cells at the junction of myofibers). Scales = 50µm.

**
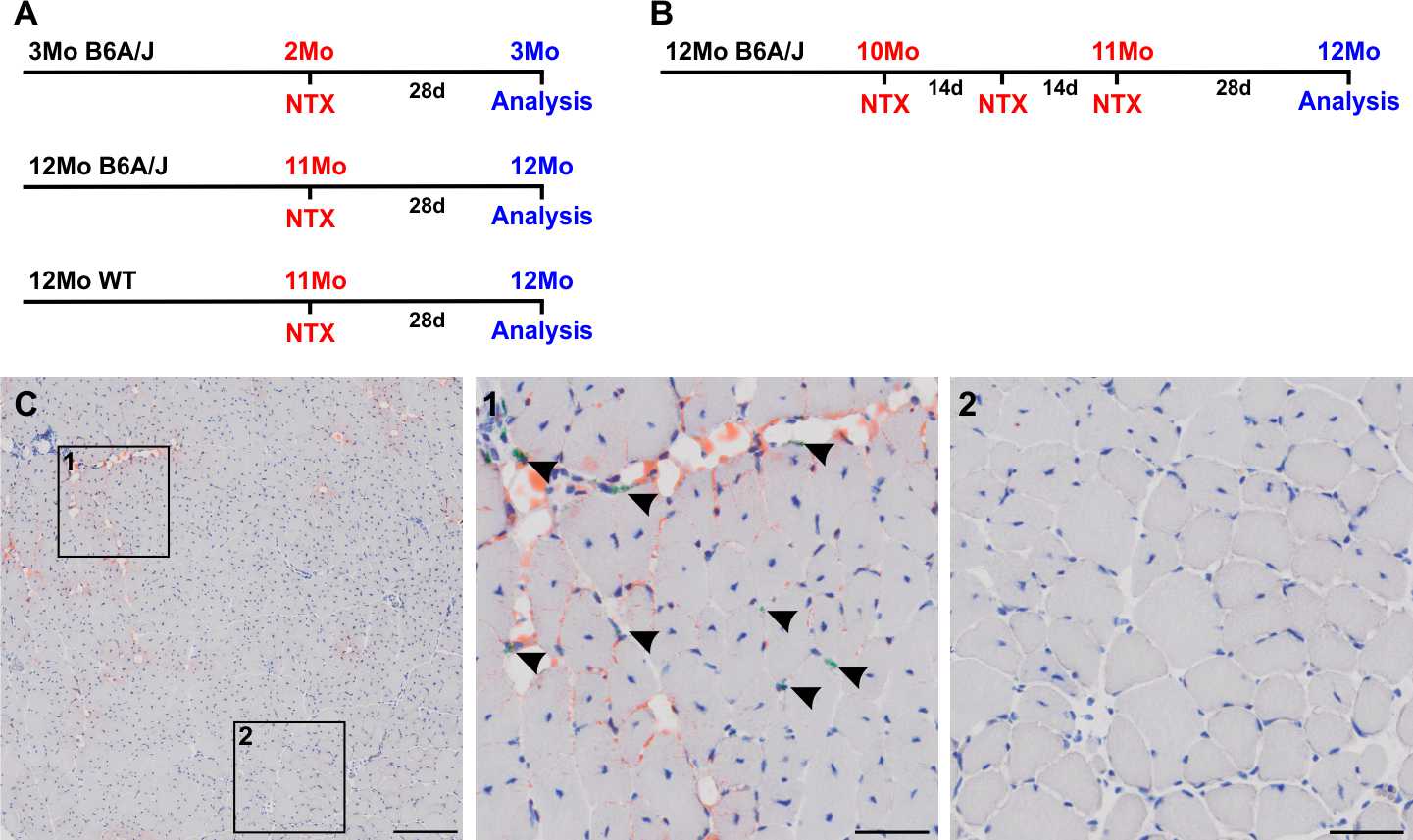
**

**Supplementary Figure 3: Notexin causes lipid deposition at the site of injury in dysferlin-deficient muscle.** (**A**) Schematic illustrating the single injury experimental procedure; a single 40µl injection of notexin (5μg/mL) was administered to the TA of B6A/J at 2 and 11Mo and 11Mo WT. Muscles were harvested 28 days later for analysis. (**B**) Schematic illustrating the repeat injury experimental procedure; B6A/J mice were injured by a series of 3 intramuscular notexin injections, each 14 days apart, from 10 to 11Mo. Muscles were harvested 28 days later for analysis. (**C**) Oil Red O staining of 12Mo B6A/J TA after a single notexin injury. Scale = 200µm. Shown are 2 regions approximately 900µm apart, which illustrate both injured and uninjured regions of the muscle. **Region 1** shows the injury site as marked by green tattoo dye (indicated by arrowheads), where centrally nucleated myofibers and Oil Red O-marked lipid are evident. **Region 2** shows an uninjured area without tattoo dye, where only spontaneous regeneration and the absence of lipid can be seen.


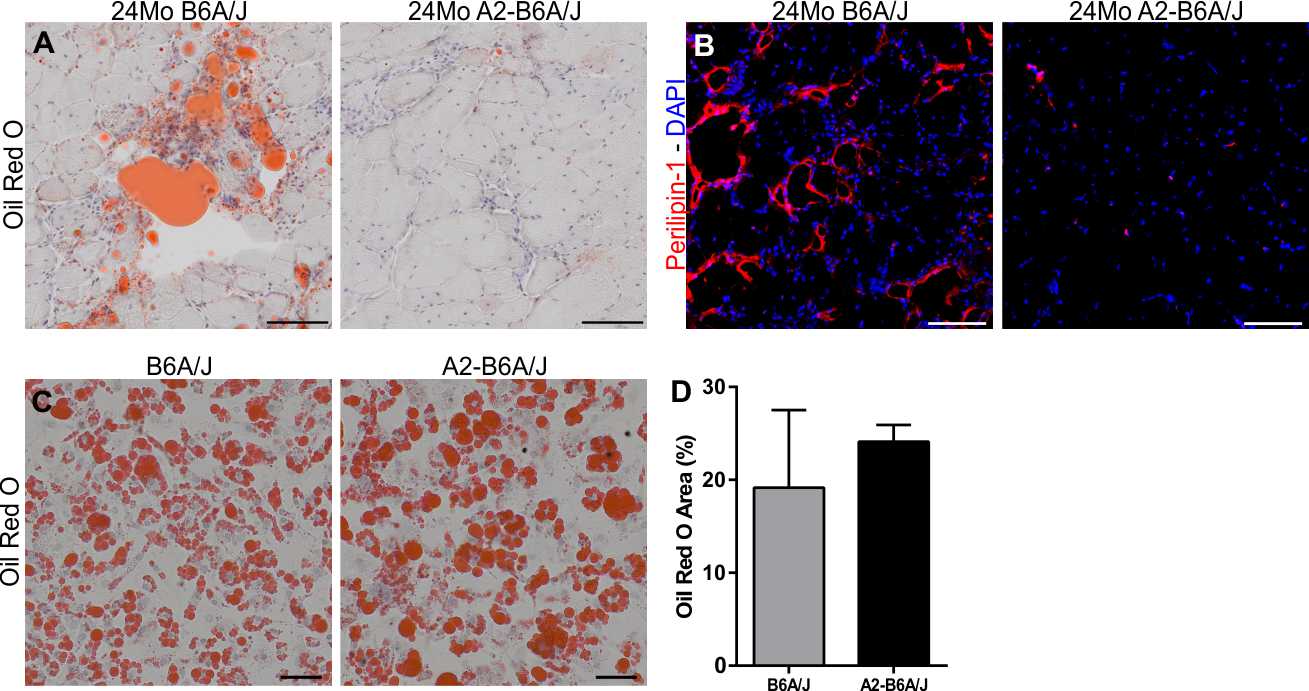


**Supplementary Figure 4: Adipogenic conversion is blocked in A2-B6A/J muscle.** (**A**) Oil Red O and (**B**) Perilipin-1 staining of quadriceps sections from 24Mo B6A/J and A2-B6A/J. Scales = 100µm. (**C**) Oil Red O staining of B6A/J and A2-B6A/J FAPs after adipogenic induction. Scale = 50µm. (**D**) Quantification of induced adipogenesis from 20,000 FAPs isolated from 24Mo B6A/J and A2-B6A/J. Data presented as mean ± SD.

**
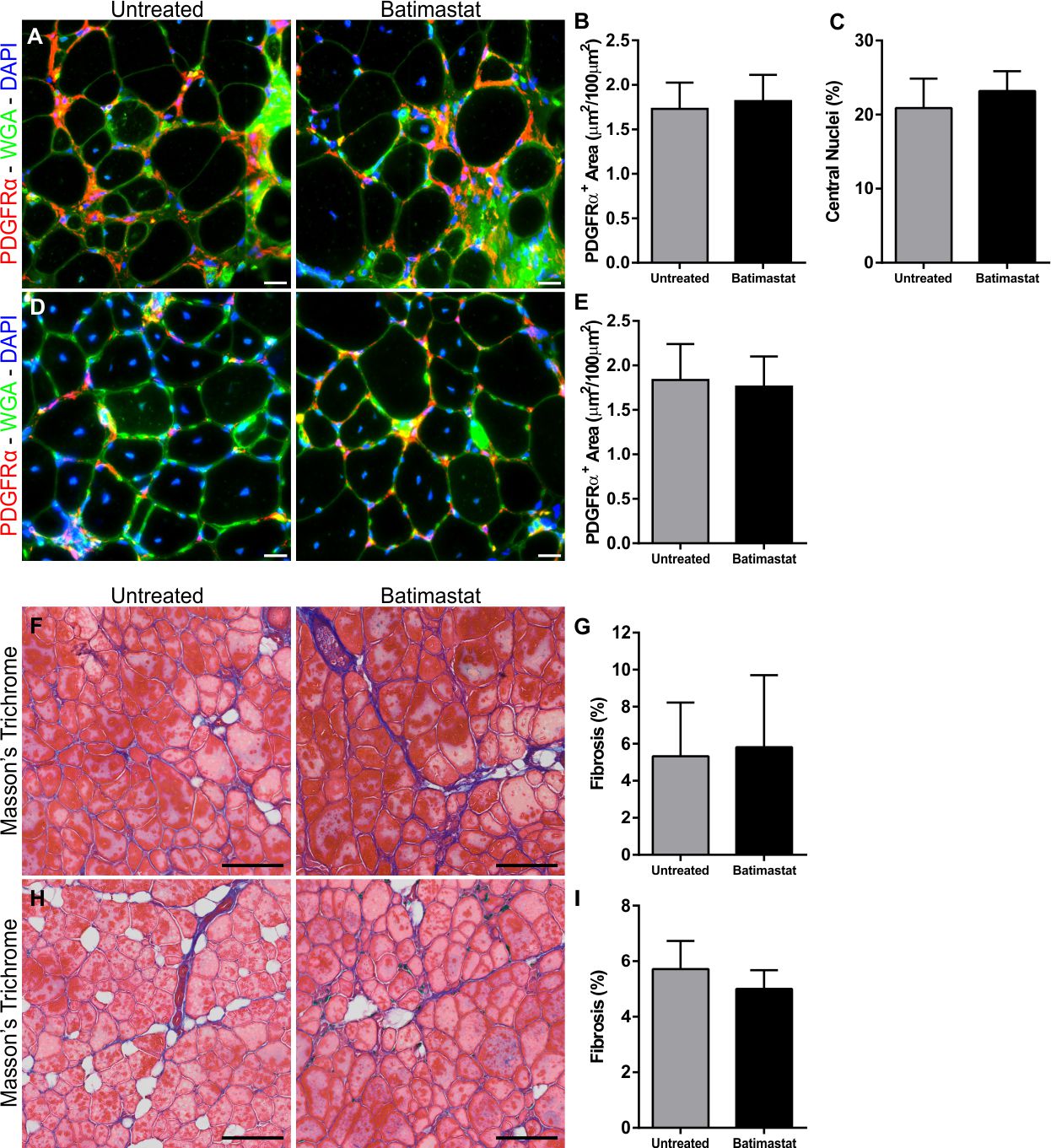
**

**Supplementary Figure 5: Batimastat does not alter accumulation of FAPs or fibrotic deposition.** (**A**) PDGFRα labelling and (**B**) quantification of PDGFRα area and (**C**) myofiber central nucleation from 14Mo gastrocnemius sections following 10 weeks of batimastat treatment. Scale = 20µm. (**D**) PDGFRα labelling and (**E**) quantification of PDGFRα area from 14Mo TA sections after 3 repeat notexin injuries and batimastat treatment. Scale = 20µm. (**F**) Masson’s trichrome staining and (**G**) quantification of fibrosis from 14Mo gastrocnemius sections following 10 weeks of batimastat treatment. Scale = 100µm. (**H**) Masson’s trichrome staining and (**I**) quantification of fibrosis area from 14Mo TA sections after 3 repeat notexin injuries and batimastat treatment. Scale = 100µm. All data displayed as mean ± SD.


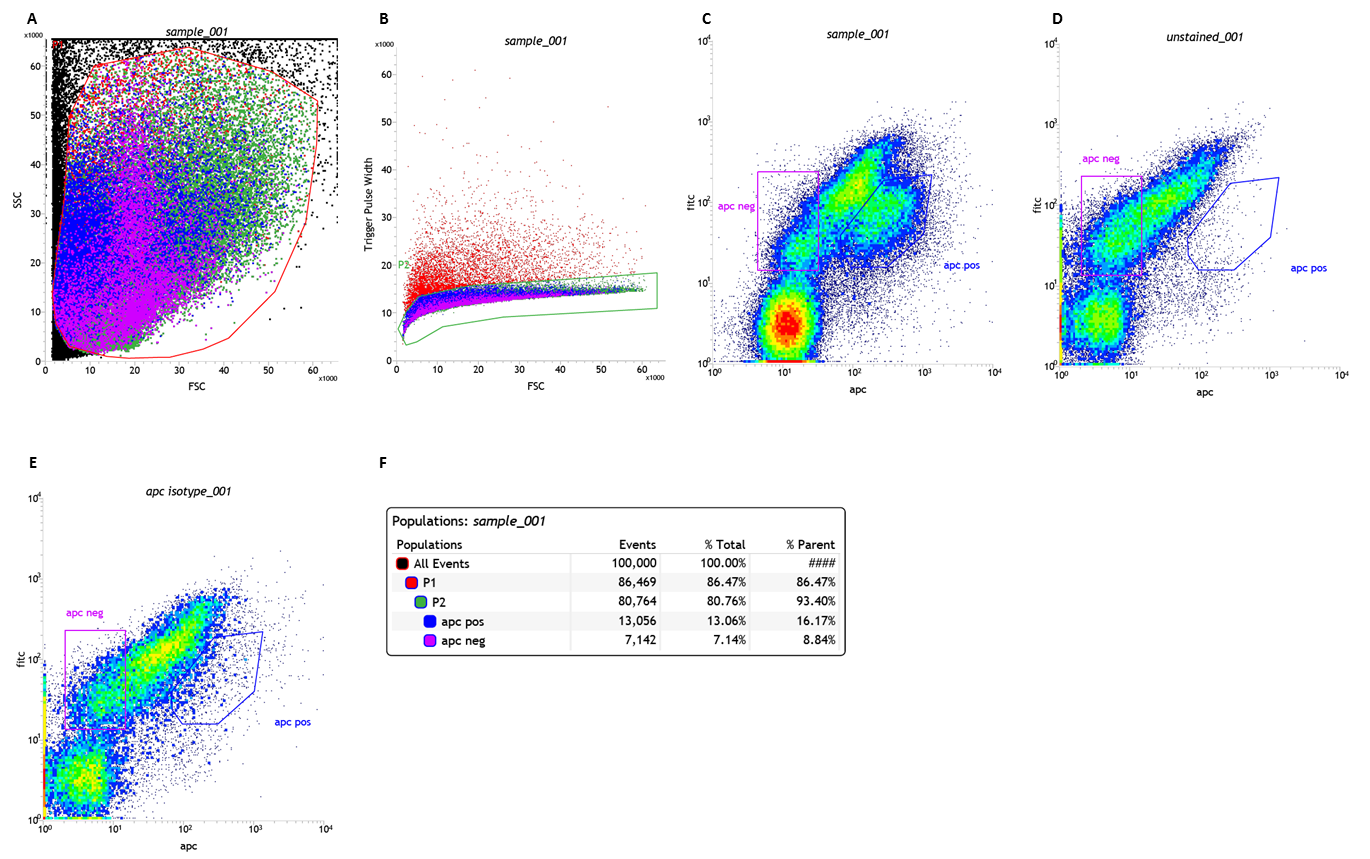
 **Supplementary Figure 6: Summary of FACS gating to enrich for FAPs.** (**A**) Cells were first gated on size by Forward Scatter (FSC) by Side Scatter (SSC) to exclude dead cells and debris. (**B**) Next, singlets were selected by gating on Forward Scatter (FSC) by the trigger pulse width. (**C**) FAPs were identified using anti-PDGFRα conjugated to APC and FITC cell autofluorescence. Positive staining was confirmed by the absence of this APC-labelled population in (**D**) unstained and (**E**) isotype controls. (**F**) PDGFRα-labelled FAPs made up 13% of the total cell suspension obtained from 12Mo B6A/J muscle. Cells negative for anti-PDGFRα were also collected to serve as controls for *in vitro* experiments.

**
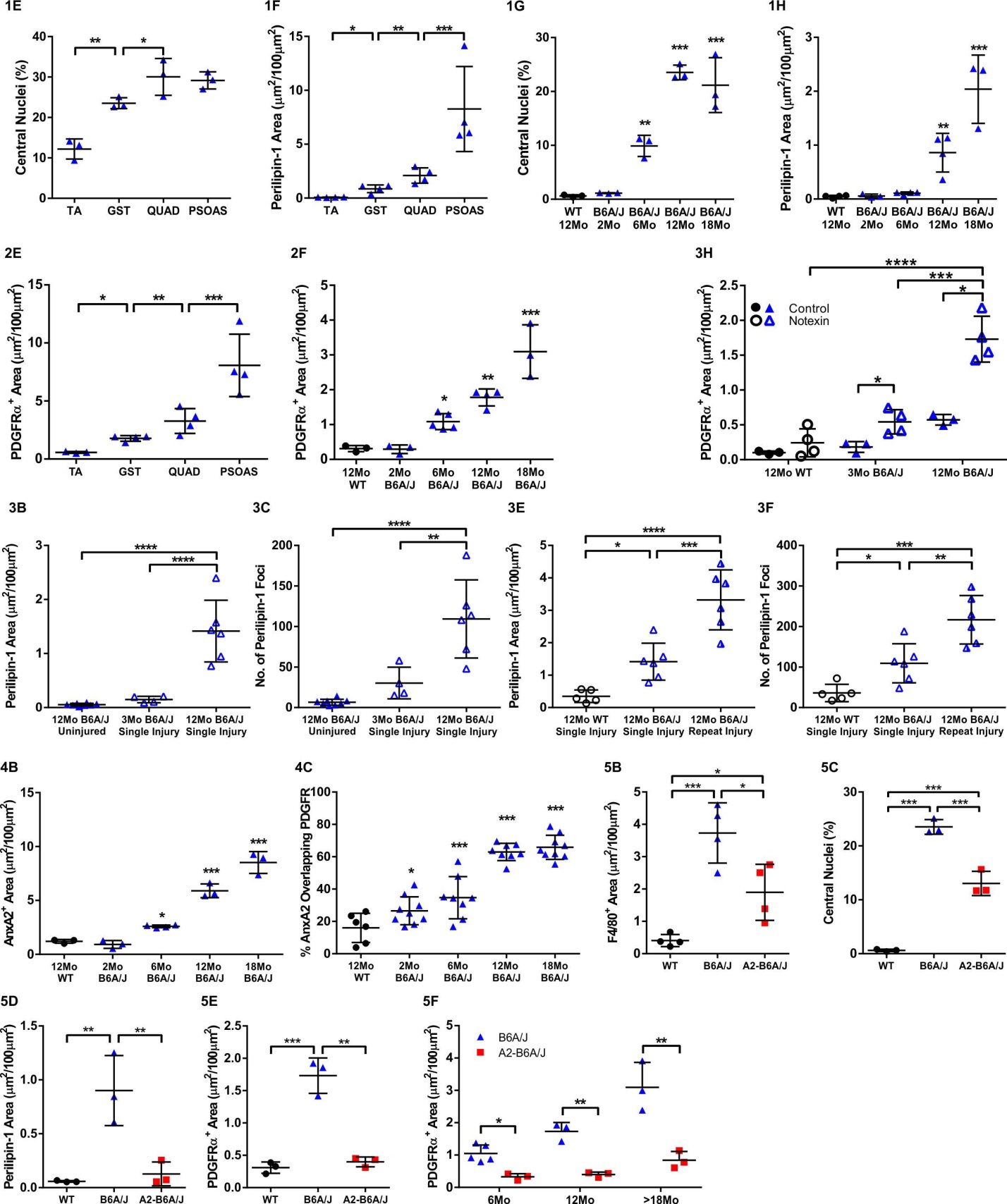
**

**Supplementary Figure 7: Illustration of data distribution.** Dot plots corresponding to the bar graphs presented in the manuscript. The figure panel labels here correspond to the bar graph presented in the main figures 1 through 5.

**
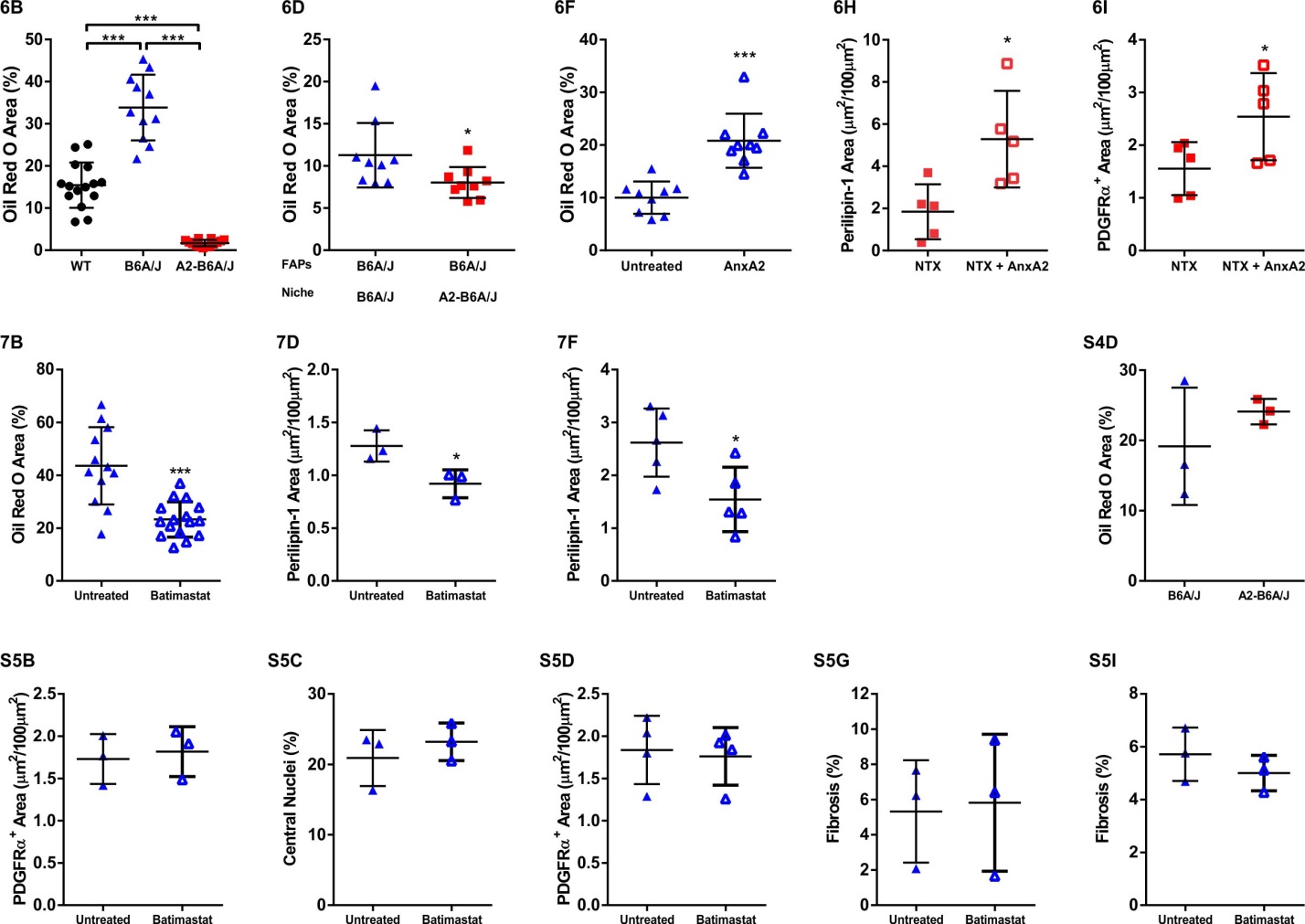
**

**Supplementary Figure 8: Illustration of data distribution.** Dot plots corresponding to the bar graphs presented in the manuscript. The figure panel labels here correspond to the bar graph presented in the main figures 6, 7 and supplementary figures.


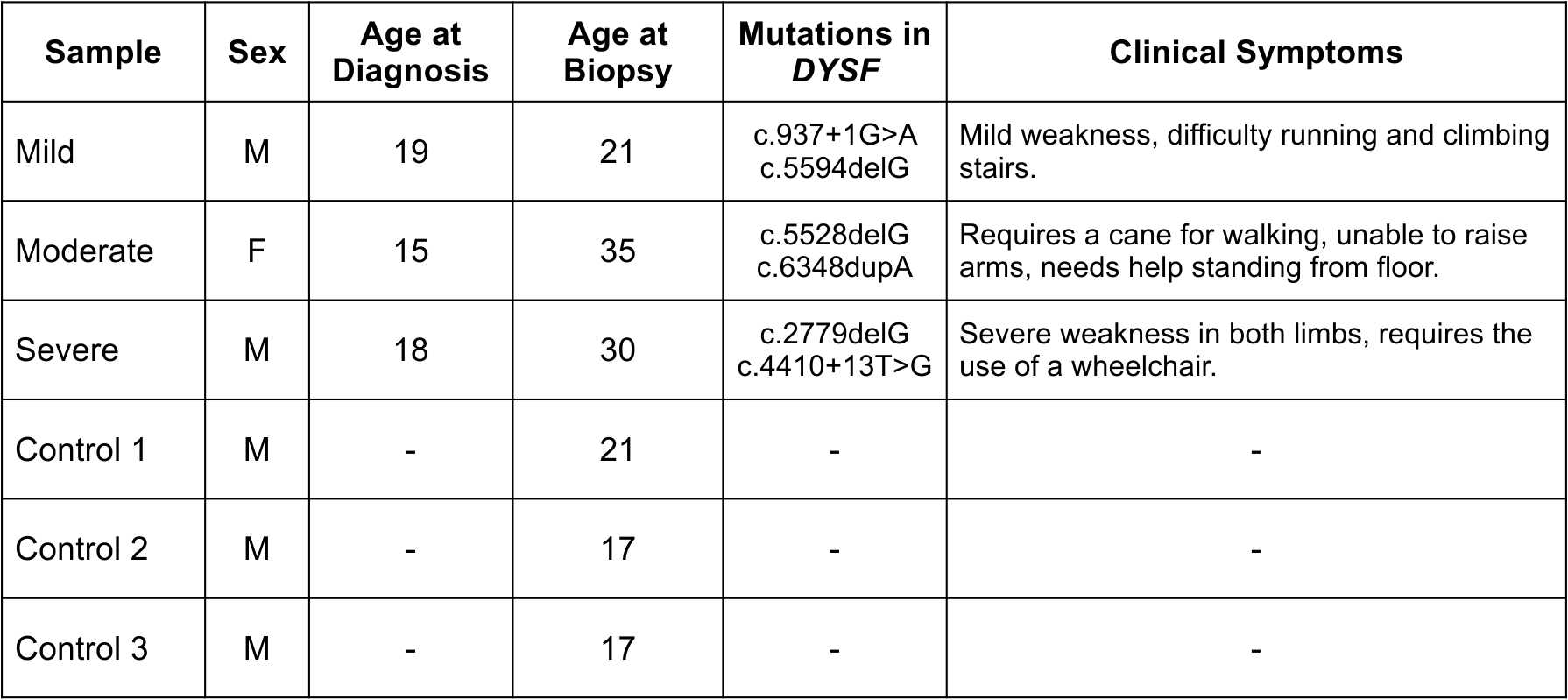


**Supplementary Table 1: Summary of LGMD2B patient biopsies included for analysis.**
