# Supplementary material for: Fibroadipogenic progenitors are responsible for muscle loss in limb girdle muscular dystrophy 2B
Source: Nat Commun. 2019 Jun 3;10:2430. doi: 10.1038/s41467-019-10438-z (PMC6547715; doi:10.1038/s41467-019-10438-z)
Supplement: Supplementary file 2 — Reporting Summary [file 41467_2019_10438_MOESM2_ESM.pdf]

nature research

Corresponding author(s): Jyoti K Jaiswal

Last updated by author(s): Apr 5, 2019

Reporting Summary

Nature Research wishes to improve the reproducibility of the work that we publish. This form provides structure for consistency and transparency in reporting. For further information on Nature Research policies, see Authors & Referees and the Editorial Policy Checklist.

Statistics

For all statistical analyses, confirm that the following items are present in the figure legend, table legend, main text, or Methods section.

n/a	Confirmed	
☐	☒	The exact sample size (*n*) for each experimental group/condition, given as a discrete number and unit of measurement
☐	☒	A statement on whether measurements were taken from distinct samples or whether the same sample was measured repeatedly
☐	☒	The statistical test(s) used AND whether they are one- or two-sided<br>*Only common tests should be described solely by name; describe more complex techniques in the Methods section.*
☒	☐	A description of all covariates tested
☒	☐	A description of any assumptions or corrections, such as tests of normality and adjustment for multiple comparisons
☐	☒	A full description of the statistical parameters including central tendency (e.g. means) or other basic estimates (e.g. regression coefficient) AND variation (e.g. standard deviation) or associated estimates of uncertainty (e.g. confidence intervals)
☐	☒	For null hypothesis testing, the test statistic (e.g. *F*, *t*, *r*) with confidence intervals, effect sizes, degrees of freedom and *P* value noted<br>*Give P values as exact values whenever suitable.*
☒	☐	For Bayesian analysis, information on the choice of priors and Markov chain Monte Carlo settings
☒	☐	For hierarchical and complex designs, identification of the appropriate level for tests and full reporting of outcomes
☒	☐	Estimates of effect sizes (e.g. Cohen's *d*, Pearson's *r*), indicating how they were calculated

Our web collection on statistics for biologists contains articles on many of the points above.

Software and code

Policy information about availability of computer code

Data collection	No custom software or computer codes were used for data collection.
Data analysis	Data were analyzed using Prism GraphPad 6 software.

For manuscripts utilizing custom algorithms or software that are central to the research but not yet described in published literature, software must be made available to editors/reviewers. We strongly encourage code deposition in a community repository (e.g. GitHub). See the Nature Research guidelines for submitting code & software for further information.

Data

Policy information about availability of data

All manuscripts must include a data availability statement. This statement should provide the following information, where applicable:

- Accession codes, unique identifiers, or web links for publicly available datasets
- A list of figures that have associated raw data
- A description of any restrictions on data availability

The authors declare that all data supporting the findings of this study are available within the paper and its supplementary information.

Field-specific reporting

Please select the one below that is the best fit for your research. If you are not sure, read the appropriate sections before making your selection.

☒ Life sciences ☐ Behavioural & social sciences ☐ Ecological, evolutionary & environmental sciences

For a reference copy of the document with all sections, see nature.com/documents/nr-reporting-summary-flat.pdf

Life sciences study design

All studies must disclose on these points even when the disclosure is negative.

Sample size	Based on our published studies, the group size of samples were determined and found to be sufficient for the assessments reported here. These sample sizes are reported in the figure legends.
Data exclusions	No data were excluded from the analyses.
Replication	A minimum of 3 biological replicates were used for all analyses.
Randomization	Mice were randomly allocated to treatment groups where applicable.
Blinding	Wherever possible investigators were blinded for genotype and/or treatment during data analysis

Reporting for specific materials, systems and methods

We require information from authors about some types of materials, experimental systems and methods used in many studies. Here, indicate whether each material, system or method listed is relevant to your study. If you are not sure if a list item applies to your research, read the appropriate section before selecting a response.

Materials & experimental systems

n/a	Involved in the study	
☐	☒	Antibodies
☒	☐	Eukaryotic cell lines
☒	☐	Palaeontology
☐	☒	Animals and other organisms
☐	☒	Human research participants
☒	☐	Clinical data

Methods

n/a	Involved in the study	
☒	☐	ChIP-seq
☐	☒	Flow cytometry
☒	☐	MRI-based neuroimaging

Antibodies

Antibodies used	Anti-Perilipin (Sigma, #P1873), Anti-PDGFRα (Cell Signaling, #3174S), Anti-Annexin A2 (Santa Cruz, #SC-9061), Anti-F4/80 (Serotec, #MCA497), Anti-PDGFRα (R&D Systems, #AF1062)
Validation	All antibodies used in this study are commercially available and have been validated by the respective companies or previously published literature and care was taken to select antibodies that were previously validated for FAPs Anti-Perilipin and Anti-PDGFRα (Uezumi et al, Nat. Cell Bio. 2010), Annexin A2 (Defour et al, Hum. Mol. Gen. 2017) and Anti-F4/80 (Novak et al, Nat. Comms 2017).

Animals and other organisms

Policy information about studies involving animals; ARRIVE guidelines recommended for reporting animal research

Laboratory animals	C57BL/6J (WT) and B6.A-Dysfprmd/GeneJ (B6A/J) mice were obtained from the Jackson Laboratory (Bar Harbor, ME), A2-B6A/J mice were generated in house as part of our previous study (Defour et al, Hum. Mol. Gen. 2017). Mice were used at the timepoints indicated from 3 - 24Mo.
Wild animals	This study did not involve wild animals
Field-collected samples	This study did not involve field-collected samples.
Ethics oversight	All animal procedures were conducted in accordance with guidelines for the care and use of laboratory animals as, and were approved by the local Children's National Medical Center Institutional Animal Care and Use Committee.

Note that full information on the approval of the study protocol must also be provided in the manuscript.

Human research participants

Policy information about studies involving human research participants

Population characteristics	Frozen muscle biopsies from LGMD2B patients with 2 confirmed mutations in dysferlin were used in the study. As a control, frozen muscle biopsies were obtained from young adults with no known neuromuscular conditions and without any histopathological features to serve as a comparison. Full descriptions of all biopsies used are provided in the manuscript.

Recruitment	Frozen muscle sections were obtained from bio-banked biopsies taken from LGMD2B patients with 2 confirmed mutations in dysferlin. Control muscle sections were obtained from bio-banked biopsies taken from young adults with no known neuromuscular conditions aged-matched as closely to the disease samples as possible.
Ethics oversight	Patient biopsies were obtained under informed consent and was approved by the Ethics Committee of Hospital de la Santa Creu i Sant Pau de Barcelona.

Note that full information on the approval of the study protocol must also be provided in the manuscript.

Flow Cytometry

Plots

Confirm that:

☒ The axis labels state the marker and fluorochrome used (e.g. CD4-FITC).

☒ The axis scales are clearly visible. Include numbers along axes only for bottom left plot of group (a 'group' is an analysis of identical markers).

☒ All plots are contour plots with outliers or pseudocolor plots.

☒ A numerical value for number of cells or percentage (with statistics) is provided.

Methodology

Sample preparation	A primary cell suspension from mouse skeletal muscle was obtained by collagenase/dispase digestion to yield single cells.
Instrument	(Becton Dickinson Influx Cell Sorter (#646500)
Software	Analysis done using BD FACS Software
Cell population abundance	PDGFRa-labelled FAPs comprise ~13% of the total primary cell suspension isolated from B6A/J skeletal muscle, but <5% in WT muscle. Purity was checked by re-analyzing the post sort population, which confirmed >95% enrichment for FAPs.
Gating strategy	Cells were first gated on size by Forward Scatter (FSC) by Side Scatter (SSC). Next, singlets were selected by gating on Forward Scatter (FSC) by the trigger pulse width. Next, cells were gated for CD140a APC positive and APC negative signals using unstained and isotype controls to identify positive signal events which were distinct from background autofluorescence on the FITC channel.

☒ Tick this box to confirm that a figure exemplifying the gating strategy is provided in the Supplementary Information.
